# Nutrient Composition and Antioxidant Performances of Bread-Making Products Enriched with Stinging Nettle (*Urtica dioica*) Leaves

**DOI:** 10.3390/foods10050938

**Published:** 2021-04-25

**Authors:** Annalisa Maietti, Paola Tedeschi, Martina Catani, Claudia Stevanin, Luisa Pasti, Alberto Cavazzini, Nicola Marchetti

**Affiliations:** Department of Chemistry, Pharmaceutical and Agricultural Sciences, University of Ferrara, via L. Borsari, 46-44121 Ferrara, Italy; mttnls@unife.it (A.M.); paola.tedeschi@unife.it (P.T.); martina.catani@unife.it (M.C.); claudia.stevanin@unife.it (C.S.); luisa.pasti@unife.it (L.P.); alberto.cavazzini@unife.it (A.C.)

**Keywords:** *Urtica dioica*, stinging nettle, enriched bread, minerals, dietary fibers, antioxidants

## Abstract

Stinging nettle (*Urtica dioica*) is an edible plant, well-known for its nutritional and nutraceutical properties. Stinging nettle leaves are typically rich in fibers, minerals and vitamins, as well as antioxidant compounds, i.e., polyphenols and carotenoids. Due to these reasons, since ancient times stinging nettle has been widely used in Italy as an ingredient in foods and beverages as a therapeutic agent. This work provides an investigation focused on bread enrichment with nettle leaves and the improvement of bread proximate composition in minerals, fibers and antioxidant compounds during product preparation. The comparison between plain and nettle enriched white bread shows a significant increase in fibers and nutrients, i.e., calcium and copper levels. Nettle enrichment also provides an increase in lutein and β-carotene, as well as in total phenols and antioxidant activity. These last two nutritional elements are remarkably high in enriched bread and it has been found that phenolic concentration increases during breadmaking steps, from kneading to primary dough fermentation and from secondary fermentation of shaped loaves to baking.

## 1. Introduction

Obesity and correlated diseases (i.e., cardiovascular diseases, diabetes and hypertension), have been grown in the last decades not only in industrialized countries but also in low and middle income countries. If the trend continues, by 2030 an estimated 38% of the world’s adult population will be overweight, and another 20% will be obese [1]. Globalization has had profound effects on lifestyle that are linked with diet, activity and subsequent imbalance that have led to the current obesity epidemic. Westernization of the world’s diet has involved reduction in fiber, whole grains, fruits and vegetables intake and an increase in refined carbohydrates and saturated fats [2]. Changes in eating patterns have resulted in an increase of energy dense meals and suboptimal nutrients intake, compared to the Recommended Daily Allowance (i.e., RDA). People who eat very few fruits and vegetables are likely to have an inadequate intake of many micronutrients, such as folic acid and vitamin C, and a consequent increment of the risks related to DNA damage, like cancer and other degenerative diseases. In addition, dietary deficiencies of micronutrients that are not derived primarily from fruits and vegetables, such as zinc, iron, and the vitamins E, niacin, B6 and B12, also seem to contribute to raise the risk of DNA damage [3].

Nowadays, food behavior begins to reverse the negative trends with major attention in health promotion. Consumers are increasingly concerned about their health and pay more attention to their lifestyle and the healthiness of their diet. The number of functional foods on the market is growing rapidly [4], and this trend looks set to continue in the future as well [5], with a growth rate of about 8–16% per year. Bread and bakery products are among the most consumed food in the world every day. They can be used as carriers to increase healthy compounds intake such as minerals, vitamins and antioxidants. Spices [6,7], marine foods [8], processing by-products of plant foods [9,10], cereals by products [11] and omega-3 fatty acids [12] are some of the ingredients that could be included in bread formulation. *Urtica dioica* is a wild comestible plant and it has been served as food since ancient times, in particular for salad, seasoning, soups and herbal infusions [13,14]. Stinging nettle is an important source of minerals and vitamins, including calcium, iron, magnesium, phosphorus, potassium, sodium, ascorbic acid and B-complex. It also contains a relevant amount of proteins, chlorophylls and carotenoids, such as β-carotene and lutein. Stinging nettle leaves are also rich in polyphenols, mainly flavonoids (i.e., kaempferol, isorhamnetin, quercetin, and their rutinoside or glycoside derivatives) and phenolic acids (i.e., caffeic acid and its ester derivatives, like chlorogenic acid and caffeoylmalic acid) [15].

In some recent works [16,17], polyphenols and carotenoids in stinging nettle leaves were determined in a quantitative way, and both the bioaccessibility and bioavailability of micronutrients and plant metabolites from enriched foods were investigated by means of in-vitro gastro-intestinal simulated digestion. The addition of dried, powdered nettle leaves to fresh egg pasta dough can increase both polyphenols and carotenoids intake, and the functional properties of this enriched food can be effectively improved with favorable fallouts to prevent the adverse effects of oxidative stress on biological structures and tissues. In this study, frozen chopped nettle leaves are added to bread dough with the aim to investigate the proximate composition of enriched bread vs. plain white bread during the bread-making and to improving the nutritional intake of minerals, fiber and antioxidant compounds.

## 2. Materials and Methods

### 2.1. Bread Making

The bread samples studied in this work were produced by a local firm (Vassalli bakering S.r.l, Ferrara, Italy) leader in frozen precooked bread production for large-scale retail trade and collective catering. Ingredients for the white bread were: 1 kg of flour type “00” (ash = 0.46%, moisture = 12.5%); 500 mL of water; 40 g of extra-virgin olive oil; 30 g of yeast; 20 g of NaCl; 10 g of enzyme (α and β amylase). Nettle-enriched bread was obtained by adding 100 g of frozen chopped nettle leaves (particle size 1–3 mm) to white bread ingredients during the kneading step. Frozen chopped nettle leaves were commercialized by the following industrial supplier: Tecniche Moderne Surgelati S.r.l. (Ravarino, Modena, Italy). The amount of nettle used for enriched bread was determined as the maximum quantity that does not affect at the same time the proper, efficient kneading of all ingredients and the acceptable organoleptic properties of baked enriched bread. This enabled keeping ingredients unaltered with no need to modify their weight ratio in enriched bread with respect to plain white. No major differences in stickiness and texture were evidenced between the two doughs. These unaltered mechanic properties of enriched dough, together with unchanged activities of yeast and enzymes, allow having uniform, consistent leavening for the two products. Among the organoleptic properties of baked enriched bread, particular attention has been paid to taste, since this can be negatively affected by some phytoconstituents (i.e., chlorophylls) in terms of bitterness and astringency.

Both plain and nettle enriched doughs (kneaded and fermented) were partially baked at 190 °C for 12 min and, then, frozen at −18 °C in a pilot plant. Final baked products consisted of bread rolls (white and nettle enriched) that were fully oven-cooked at 200 °C for 5 min. The bread making process of both bread type (i.e., white and nettle enriched) was repeated in triplicate, and five samples were collected and stored at −20 °C from the following phases: kneading, leavening, prebaking (i.e., partially baked bread) and baking (i.e., fully baked bread). For each phase and for each bread type the samples were homogenized and analyzed in triplicate.

### 2.2. Proximate Composition

Methods of analysis by Association of Official Analytical Chemists (AOAC, 1984 [18]) were adopted to determine sample proximate composition. Moisture content was determined by drying the samples at 105 °C to a constant weight. Moisture was expressed as g of water per 100 g of fresh weight (g/100g_fw_). Total nitrogen compounds were determined on 1 g of dry matter by technically revised Kjeldahl method by International Organization for Standardization (ISO 8968-3:2004) [19] that replaces previous IDF 20-3:1993 method by International Dairy Federation. The protein content was determined by means of a conversion factor equal to 6.25 (Kjeldahl digestion unit and Kjeldahl distillation unit made from VELP Scientifica, Usmate, MB, Italy). Ashes were determined by the incineration of 1 g of dry matter placed in a muffle furnace (VELP Scientifica, Usmate, MB, Italy) at 570 °C for 5 h. Total lipids were determined by gas-chromatography after soxhlet extraction (VELP Scientifica, Usmate, MB, Italy). Gas-chromatographic runs were done after fatty acid transesterification with 1.5 mL of 5% NaOH solution in methanol. Sample volume of 1 μL was injected into the gas chromatography–mass spectrometry (GC-MS) apparatus. MS detector was a Varian Saturn 2100 MS/MS ion trap mass spectrometer. MS data acquisitions were performed in full-scan mode. Separations were obtained by using a Zebron ZB-WAX (Phenomenex) capillary column (60 cm length, 0.25 mm i.d.) and helium as carrier gas at 1 mL/min constant flow. The injector temperature was set up at 250 °C and the oven temperature program was isothermal at 100 °C for 2 min, then temperature was increased up to 200 °C at 10 °C/min, then isothermal at 200 °C for 108 min. This was employed for fatty acids profiling in both frozen chopped nettle and enriched bread. Insoluble dietary fiber (IDF) and soluble dietary fiber (SDF) were determined as reported by the instruction protocol developed for the Megazyme Total Dietary Fiber Assay Kit (Megazyme International, Co. Wicklow, Ireland), based on the combined enzymatic and gravimetric method by Prosky et al. [20] and by Lee et al. [21]. All other carbohydrates were estimated by difference. Results were expressed as g/100g_fw_.

### 2.3. Inorganic Composition

A Perkin-Elmer (Perkin-Elmer, Inc.,Waltham, MA, USA) Atomic Absorption Spectrometer (AAS) was used for mineral element analysis, after nitric acid digestion by an acid-assisted microwave irradiation. All samples were digested in triplicate by using a published method [22]. 1 g of dry matter (dm) was placed in the microwave vessel and 5 mL of concentrated HNO_3_ were added and submitted to a digestion cycle (ramp: 200 °C in 20 min, hold for 10 min). After cooling, 1 mL of H_2_O_2_ was added and the mixture was submitted to another digestion cycle until the oxidation of the organic matter was completed. Each digested solution was diluted with ultrapure water unto a final volume of 20 mL after cooling.

### 2.4. Extraction of Phenolic Compounds

A portion of chopped nettle and each bread sample were dried at 60 °C and then ground with a knife mill Retsch GM200 (Retsch Verder Scientific S.r.l., Torre Boldone, Bergamo, Italy) at 2500 rpm for 30 s to obtain a dry powder. The extraction of phenolic compounds from samples was carried out using a method proposed by Pérez-Jiménez and Saura-Calixto [23], with some modifications [16]. Briefly, 2 g of nettle powder or 5 g of bread powders were placed in a test tube with 15 mL of a mixture of methanol/water (80:20) acidified with 0.1% of formic acid and homogenized by an T18 basic Ultraturrax (IKA^®^-Werke GmbH & Co. KG, Staufen, Germany) for 2 min. The sample was extracted three times and after each step, samples were centrifuged (Centrifuge Thermo PK121R, Thermo Fisher Scientific, Waltham, MA, USA) at 2500 g for 5 min at 4 °C, and supernatants were recovered and combined.

### 2.5. Total Phenols Content (TPC) and Antioxidant Activity (TEAC)

The amount of total phenols (TPC) in extracted samples was measured using the Folin Ciocalteu assay as described by Singleton et al. [24]. The results are expressed as µg of gallic acid equivalents per g of fresh weight (fw), µg_GAE_/g_fw_ Antioxidant activity of the extracts was measured by using the Trolox equivalent antioxidant capacity (TEAC) assay based on scavenging of 2,2′-azinobis-(3-ethylbenzothiazoline-6-sulfonic acid) radical (ABTS^•+^) as reported by Re et al. [25]. Results were expressed as mg of trolox equivalents per g of fresh weight, mg_TE_/g_fw_.

### 2.6. Extraction and Analysis of Carotenoids

Carotenoids were extracted from dried powder samples using the method developed by Panfili et al. [26], slightly modified in our laboratory [17]. About 0.15 g of nettle powder or 1.5 g of bread powders were placed in a test tube with 5 mL of Pyrogallol 60 g/L solution in Ethanol, 2 mL of Ethanol, 2 mL of NaCl 10 g/L aqueous solution and 2 mL of KOH 600 g/L aqueous solution. After homogenization with Ultraturrax (IKA^®^-Werke GmbH & Co. KG) for 1 min, the samples were shaken for 45 min at 70 °C, cooled in an ice bath for 5 min and 15 mL of NaCl (10 g/L) were added at last. Samples were extracted twice with 15 mL hexane: ethyl acetate 9:1 added by BHT 0.1% *w*/*v*. The organic phase was evaporated using a rotary evaporator and the dried extract was resuspended with 2 mL of mobile phase for HPLC analysis. The analysis of carotenoids was carried out using a previously developed method [27] based on an HPLC Agilent 1100 (Agilent Inc., Santa Clara, CA, USA) equipped with DAD detector and C30 Develosil RP-Aqueous 150 × 3.0 mm column (Phenomenex, Torrance, CA, USA). Mobile phases employed were binary mixtures of water and acetonitrile 70:30 *v*/*v* (solvent A) and methanol and methyl tert-butyl ether 50:50 *v*/*v* (solvent B). Eluent composition was programmed as in the following by changing the amount of solvent B: from 20% to 47% in 10 min, from 47% to 57% in 10 min, and then from 57% to 100% in 20 min. The flow rate was 0.4 mL/min and the injection volume was 5 µL. The spectrophotometric detection was done at 450 nm.

### 2.7. Statistical Analysis

Data are presented as means ± SD of three parallel measurements. Differences between bread samples were analyzed by ANOVA using SPSS v. 22.0 software (SPSS Inc., Chicago, IL, USA). *p* values < 0.05 was regarded as significant.

## 3. Results and Discussion

### 3.1. Chemical Composition of Frozen Chopped Nettle

In a recent literature work [28] dried nettle was compared to barley and wheat flours in terms of their main nutritional components, and results evidenced that dried nettle has a higher content of protein, fibers, fat, ash, calcium, iron, and antioxidant compounds such as tannins, total polyphenols and carotenoids than cereal flours. With regard to the uptake of metals by plants, it is already known that this is strongly influenced by different factors, including type of plant, nature of soil, climate, and agriculture practices. Also, nettle plants can selectively absorb some elements from soil, as it has been proven by a literature work where among 30 medicinal herbal samples analyzed by ICP-MS [29] the highest levels of iron, copper and zinc were found in stinging nettle. Additionally, another study has been shown a high correlation between Fe, Mn, Cu, and K concentrations in soil and their levels in the aerial part of nettle leaves [30]. The chemical composition nettle used in this study has been determined and listed in Table 1. Values are reported as grams per 100 g of fresh weight (g/100g_fw_). Nettle displayed a higher content of ashes and total dietary fiber than “00” flour normally used for bread making. The high level of mineral fraction is mainly due to Ca (6764 µg/g_fw_) and K (6456 µg/g_fw_), while Fe and Cu content were 67.63 µg/g_fw_ and 190.6 µg/g_fw_, respectively (see Table 2). Some therapeutic benefits of nettle are attributed to its secondary metabolites (phenolic compounds, carotenes, essential oil) and to its antioxidant activity [31]. TPC, antioxidant activity and concentration of lutein and β-carotene were determined; these values are reported in Table 3. The nettle sample displays the following characteristics: TPC equals 824 µg_CE_/g_fw_, 67.85 µg/g_fw_ of lutein and 5.70 µg/g_fw_ of β-carotene. The antioxidant activity determined by TEAC assay was 0.89 mg_TE_/g_fw_.

### 3.2. White vs. Enriched Nettle Bread Proximate Composition

The chemical composition of white bread and nettle enriched bread are expressed per 100 g of fresh product and are reported in Table 4. Amount of moisture, proteins and carbohydrates in white bread were similar to those in nettle bread. The addition of nettle to the dough significantly increased ashes content from 1.28 to 1.75 g/100g_fw_ and dietary fibers content from 0.57 to 1.22 g/100g_fw_. Conversely, amount of fats decreased (*p* < 0.05) from 2.51 to 2.13 g/100g_fw_ between white and enriched bread. Results are in agreement with other literature findings [32], where nettle powder was used to enrich other foods but with reduced sensory acceptability, i.e., noodles that are higher in proteins, ashes and fibers. In our study, the enrichment with nettle produces only slight modifications on organoleptic characteristics of white bread (i.e., color and flavor), but it does not increase the content of proteins.

The mineral composition of ashes was investigated by atomic spectroscopy and the results obtained are reported in Table 5. Ingredients used for white and enriched bread were the same and the observed increment was only due to nettle added. Sodium display the higher amount because 20 g of salt have been added to both bread types. Also, potassium was determined at high concentrations (1457 and 1877 µg/g_fw_ in white and nettle enriched bread, respectively). A significant increment of calcium, copper and a moderate increment (*p* < 0.05) of iron were observed in nettle enriched bread. After nettle addition, calcium concentration increases about four times (from 126 µg/g_fw_ to 559 µg/g_fw_) while copper concentration rises roughly eight times (from 2.35 µg/g_fw_ to 16.51 µg/g_fw_). RDA for calcium is set at 800–1200 mg/day for adults in Italy, and it has been reported [33] that Ca intake with an Italian diet only allows for meeting 76% of the RDA value. This confirms the trend evidenced by other studies performed in different European countries. Assuming a bread intake of 100 g/day, the nettle enrichment bread supplies the 5–7% of Ca RDA toward 1.05–1.58 of the white bread. Copper and iron are trace elements involved in cell oxidation and signaling systems. They act as cofactors in several enzymatic reactions that play a critical role in several metabolic pathways. The Italian RDAs are 900 µg/day for Cu and 10–15 mg/day for iron in adults. The intake of copper determined in the Italian diet is estimated to be high enough [33]; however, the human gastrointestinal system can absorb only 30–40% of ingested copper from the diet [34]. In the Italian diet the daily iron intake is 12.7 mg, of which about 89% is non-haem iron [33]. Absorption of non-haem iron is usually lower than 10% [35]. The consumption of 100 g/day of enrichment bread supplies the 10–15% of RDA for Fe and 74% for Cu.

For the sake of completeness, fatty acids profile for both frozen chopped nettle and enriched bread revealed that nettle mainly contains (relative amount on a fresh matter basis) 26.1% ± 0.4% Palmitic (C16:0), 28.2% ± 1.9% Linoleic (C18:2), 22.1% ± 1.9% Linolenic (C18:3) and 13.0% ± 1.1% Oleic (C18:1), 6.1% ± 0.5% Stearic (C18:0) and 2.4% ± 1.0% Arachidic (C20:0). Enriched bread, instead, displays the same lipidic profile of extra-virgin olive oil added to the dough: 71.8% ± 0.1% Oleic (C18:1), 12.2% ± 0.2% Palmitic (C16:0), 9.7% ± 0.1% Linoleic (C18:2) and 4.6% ± 0.1% Stearic (C18:0). No differences were evidenced between white bread and nettle enriched bread regarding fatty acid profile.

### 3.3. Influence of Nettle Addition on Antioxidant Compounds of Bread

Antioxidant activity of plants have been associated with their ability to prevent disorders like cancer, diabetes, cardiovascular diseases, autoimmune diseases, neurodegenerative disorders and aging. Most of the antioxidant activity of plants is due to the presences of secondary metabolites like polyphenols and carotenoids. On one hand, bread wheat (*Triticum aestivum* subsp. *aestivum*) contains very limited quantities of carotenoids and phenolics. In the whole grain, these compounds exist mainly in the bran and germ portions, where they are covalently cross-linked with cell wall carbohydrate polymers. Starchy endosperm is separated from bran and germ portions by milling process and only the first fraction produces four, whereas the last two are commonly discarded and used for animal feeding [36,37]. On the other hand, nettle leaves (*Urtica dioica* variety) are rich in phyto-constituents, mainly polyphenols, flavonoids (kaempferol, isorhamnetin, quercetin, isoquercitrin and rutin) and phenolic acids (caffeic acid and chlorogenic acid), and carotenoids (β-carotene, hydroxyl-β-carotene, luteoxanthin, lutein epoxide, and violaxanthin), but also essential oils, fatty acids and other constituents (minerals and vitamins) [31].

Many studies have been conducted on the topic of bread enrichment with pseudo-cereals [38] or with spices, herbs and the green edible part of plants [39,40]. In this context, it is interesting to study the antioxidant properties of nettle enriched bread. Thus, antioxidant activity, polyphenols, β-carotene and lutein contents of white and nettle enriched breads are assayed in this work and they are reported in Table 6. TPC value increases from 372 µg_GAE_/g_fw_ in white bread to 597 µg_GAE_/g_fw_ in nettle enriched one. Nettle leaves contain plenty of antioxidant compounds and those used in this study have been characterized (see Table 3). As expected, the antioxidant activity is higher in enriched bread than in plain white (i.e., 0.83 vs. 0.53 mg_TE_/g_fw_, respectively). Therefore, nettle addition provides a significant increase of total phenols and an increment (*p* < 0.05) of antioxidant activity.

β-carotene and lutein are quantitatively determined by using external calibration method. Area vs. concentration data have been fitted to a linear regression model and correlation coefficients were 0.9969 for β-carotene and 0.9987 for lutein. Nettle addition to bread significantly improves (*p* < 0.05) β-carotene and lutein contents from 0.412 µg/g_fw_ to 0.723 µg/g_fw_ and from 1.46 µg/g_fw_ to 3.97 µg/g_fw_, respectively. Raw chopped nettle is used to enrich white bread displayed lutein and β-carotene contents of 67.8 µg/g_fw_ and 5.70 µg/g_fw_, respectively (see Table 3). If we consider that this ingredient is added in terms of 10% w/w to bread dough, we would have expected higher values for both carotenoids then what we have found (i.e., 8.245 vs. 3.97 µg/g_fw_ for lutein and 0.982 vs. 0.31 µg/g_fw_ for β-carotene). Carotenoids are lipophilic molecules that are sensitive to thermal and enzymatic degradation, which may explain the lower content of β-carotene and lutein observed after bread additivation. Wheat contains enzymes such as lipoxygenase and peroxidase which become active when water is added to flour and can lead to oxidation of carotenoid pigments [41]. Moreover, carotenoids degradation may be mostly related to their susceptibility to heat during baking [42].

All chemical parameters determined and discussed in terms of proximate composition (i.e., inorganic elements, ashes, proteins) have been estimated for both white and nettle bread and for intermediate products during the baking process (kneading fermentation, prebaking, baking). However, these latter findings are not reported because no significant differences were found. The increase of these values with bread baking is low and most likely due to the loss of water during the cooking procedure. The breadmaking process consists of three basic operations: mixing, fermentation and baking. Some studies show how the antioxidant potential in bakery products is connected to manufacturing formulation and conditions. In particular, the antioxidant activity and residual content of free phenolic acids of flour were reduced by mixing, but increased by fermentation and baking [43]. A microbial hydrolysis reaction takes place during fermentation and this is probably responsible for the greater content of phenolic compounds and flavonoids. It is widely reported in literature that fermentation induces the breakdown of bran cell wall structure, and this leads to the release of bound phenolic compounds and to a slight increase of TPC [44]. Another hypothesis deals with chemical changes in phytocompounds or conversion of glycosylated phenolics into their aglycones. As evidenced by results in Table 7, yeast fermentation allows a total phenols increase from 706 µg_GAE_/g_dw_ in kneaded bread dough to 777 µg_GAE_/g_dw_ in fermented one. Conversely, the change in antioxidant activity is not statistically relevant.

Additionally, there are numerous evidences in literature works about an increase in TPC value due to both baking process and the concurring Maillard reaction [43,45]. Indeed, our results (Table 7) show the increase of TPC from 777 µg_GAE_/g_dw_ in fermented bread dough to 835 µg_GAE_/g_dw_ in fully baked bread, and at the same time TEAC raises from 0.79 mg_TE_/g_dw_ to 1.16 mg_TE_/g_dw_. Both TPC and TEAC do not significantly change between fermented dough and pre-baked stages. These findings can be manly due by the release of antioxidant compounds and the loss of water during cooking procedure: as a matter of fact the relative humidity dropped from 39.9% for kneaded bread to 28.5% for baked bread. For this reason TPC and TEAC quantities are calculated on the basis of dry weight and their final estimates undoubtedly confirm that phenolic content and antioxidant activity of white bread can be improved by both nettle enrichment and breadmaking process.

These results are preliminary but fundamental for a clearer understanding of the entire enrichment process and its future perspectives in functional food research. What we have found and discussed here will be applied in the following to deeply investigate the true effects of enriched foods on gastrointestinal system and human health. Staple enriched food as a delivery medium for micronutrients, dietary fibers and antioxidants represents one of the most effective ways of achieving healthier foodstuff and counteracting nutritional deficiencies in population groups (i.e., children, elders, people affected by gastrointestinal diseases).

## 4. Conclusions

In this study the comparison between white bread and nettle-enriched bread was carried out with a major focus on the chemical composition of both processed foods and with particular attention to those functional characteristics that improve the quality of nettle-enriched bread. It has been demonstrated that the addition of nettle can significantly increase the level of fibers, calcium and copper, and in a lesser way also the iron content. Nettle-enriched bread evidenced a total phenolic content higher than plain white bread and its exhibited antioxidant activity is superior in the same way than non-enriched bread. This went along with an increment in the concentration of lutein and β-carotene, two important carotenoids involved in free-radical scavenging activity. Changes in all these parameters were monitored during main breadmaking steps for large scale retail trade. The most interesting results were obtained for total phenols and antioxidant activity in enriched bread. In fact, values of TPC and TEAC increased from kneaded to fermented dough and then again moved up when fermented dough was baked.

Our findings confirm that the nettle leaves are a valuable ingredient for the development of enriched foods with improved nutritional and functional properties. This can be done on a large scale production such as breadmaking. Additionally, the enhanced antioxidant features of enriched bread might exert interesting nutritional benefits, such as protective effects with respect the mitochondrial gene expression in the presence of cereal mycotoxins. This represents a driver for future research in this field, as recently evidenced from the role of dietary carotenoids against aflatoxins and enniatins [46].

## Figures and Tables

**Table 1:** Proximate composition of frozen chopped nettle.

Component		g/100g_fw_^1^
Moisture	-	79.8 ± 0.6
Proteins	-	3.50 ± 0.02
Fats	-	0.17 ± 0.04
Ashes	-	3.38 ± 0.17
Carbohydrates	-	5.03 ± 0.28
Total fiber	-	8.15 ± 0.27
-	Soluble	1.55 ± 0.24
-	Insoluble	6.60 ± 0.51

^1^ values are expressed as mean ± SD, *n* = 3.

**Table 2:** Ashes composition of frozen chopped nettle.

Component	(µg/g_fw_) ^1^
Calcium	6764 ± 220
Potassium	6456 ± 163
Magnesium	845.4 ± 7.1
Copper	190.6 ± 8.1
Iron	67.63 ± 0.57
Sodium	32.25 ± 5.16
Zinc	6.920 ± 1.150
Manganese	11.80 ± 0.37

^1^ values are expressed as mean ± SD, *n* = 3.

**Table 3:** Antioxidant compounds in frozen chopped nettle.

Component	Nettle ^1^
TPC (µg_GAE_/g_fw_) ^2^	755 ± 21
TEAC (mg_TE_/g_fw_) ^3^	0.89 ± 0.07
Lutein (µg/g_fw_)	67.8 ± 4.1
β-carotene (µg/g_fw_)	5.70 ± 0.59

TPC, total phenols content. TEAC, antioxidant activity. ^1^ values are expressed as mean ± SD, *n* = 3. ^2^ µg of gallic acid equivalents (GAE) per g of fresh weight (fw). ^3^ mg of trolox equivalents (TE) per g of fresh weight (fw).

**Table 4:** Proximate composition of white bread and nettle enriched bread.

Component	White Bread ^1^	Nettle Enriched Bread ^1^
Moisture	26.85 ± 2.67	28.46 ± 2.46
Proteins	9.50 ± 0.61	8.97 ± 0.01
Fats *	2.51 ± 0.06	2.13 ± 0.03
Ashes *	1.28 ± 0.01	1.75 ± 0.04
Carbohydrates	60.34 ± 1.35	58.47 ± 2.37
Total fiber *	0.53 ± 0.03	1.22 ± 0.09

^1^ values are expressed as g/100g_fw_ and reported as mean ± SD, *n* = 3. * significantly different at *p* < 0.05.

**Table 5:** Ashes composition of white bread and nettle enriched bread.

Component	White Bread ^1^	Nettle Enriched Bread ^1^
Calcium *	126 ± 8	559 ± 27
Magnesium	181 ± 10	245 ± 19
Potassium	1457 ± 58	1877 ± 192
Sodium	7384 ± 597	6928 ± 371
Copper *	2.35 ± 0.12	16.51 ± 0.61
Iron *	11.77 ± 0.26	15.26 ± 1.00
Zinc	7.26 ± 0.42	7.00 ± 0.28
Manganese	4.35 ± 0.25	5.32 ± 0.21

^1^ values are expressed as µg/g_fw_ and reported as mean ± SD, *n* = 3. * significantly different at *p* < 0.05.

**Table 6:** Antioxidant compounds of white bread and nettle enriched bread.

Component	White Bread ^1^	Nettle Enriched Bread ^1^
TPC * (µg_GAE_/g_fw_) ^2^	372 ± 14	597 ± 17
TEAC (mg_TE_/g_fw_) ^3^	0.53 ± 0.07	0.83 ± 0.08
Lutein * (µg/g_fw_)	1.46 ± 0.11	3.97 ± 0.54
β-carotene * (µg/g_fw_)	0.41 ± 0.07	0.72 ± 0.10

^1^ values are expressed as mean ± SD, *n* = 3. ^2^ µg of gallic acid equivalents (GAE) per g of fresh weight (fw). ^3^ mg of trolox equivalents (TE) per g of fresh weight (fw). * significantly different at *p* < 0.05.

**Table 7:** Phenol content and antioxidant activity in nettle enriched bread production steps.

Bread Sample	TPC ^1^ (µg_GAE_/g_dw_) ^2^	TEAC ^1^ (mg_TE_/g_dw_) ^3^
Kneaded	706 ± 11	0.80 ± 0.07
Fermented	777 ± 15	0.79 ± 0.10
Pre-baked	779 ± 9	0.72 ± 0.06
Baked	835 ± 23	1.16 ± 0.10

TPC, total phenols content. TEAC, antioxidant activity.^1^ values are expressed as mean ± SD, *n* = 3. ^2^ µg of gallic acid equivalents (GAE) per g of dried weight (dw). ^3^ mg of trolox equivalents (TE) per g of dried weight (dw).

## Data Availability

Not applicable.
